# Microbial inoculation in rice regulates antioxidative reactions and defense related genes to mitigate drought stress

**DOI:** 10.1038/s41598-020-61140-w

**Published:** 2020-03-16

**Authors:** Dhananjaya P. Singh, Vivek Singh, Vijai K. Gupta, Renu Shukla, Ratna Prabha, Birinchi K. Sarma, Jai Singh Patel

**Affiliations:** 1 ICAR-National Bureau of Agriculturally Important Microorganisms, Kushmaur, Maunath Bhanjan 275101 India; 2 Department of Mycology and Plant Pathology, Institute of Agricultural Sciences, Banaras Hindu University, Varanasi, 21005 India; 3 Department of Chemistry and Biotechnology, School of Science, Tallinn University of Technology, Akadeemia tee 15, 12618 Tallinn, Estonia

**Keywords:** Plant signalling, Drought

## Abstract

Microbial inoculation in drought challenged rice triggered multipronged steps at enzymatic, non-enzymatic and gene expression level. These multifarious modulations in plants were related to stress tolerance mechanisms. Drought suppressed growth of rice plants but inoculation with *Trichoderma*, *Pseudomonas* and their combination minimized the impact of watering regime. Induced *PAL* gene expression and enzyme activity due to microbial inoculation led to increased accumulation of polyphenolics in plants. Enhanced antioxidant concentration of polyphenolics from microbe inoculated and drought challenged plants showed substantially high values of DPPH, ABTS, Fe-ion reducing power and Fe-ion chelation activity, which established the role of polyphenolic extract as free radical scavengers. Activation of superoxide dismutase that catalyzes superoxide (O_2_^−^) and leads to the accumulation of H_2_O_2_ was linked with the hypersensitive cell death response in leaves. Microbial inoculation in plants enhanced activity of peroxidase, ascorbate peroxidase, glutathione peroxidase and glutathione reductase enzymes. This has further contributed in reducing ROS burden in plants. Genes of key metabolic pathways including phenylpropanoid (*PAL*), superoxide dismutation (*SODs*), H_2_O_2_ peroxidation (*APX*, *PO*) and oxidative defense response (*CAT*) were over-expressed due to microbial inoculation. Enhanced expression of *OSPiP* linked to less-water permeability, drought-adaptation gene *DHN* and dehydration related stress inducible *DREB* gene in rice inoculated with microbial inoculants after drought challenge was also reported. The impact of *Pseudomonas* on gene expression was consistently remained the most prominent. These findings suggested that microbial inoculation directly caused over-expression of genes linked with defense processes in plants challenged with drought stress. Enhanced enzymatic and non-enzymatic antioxidant reactions that helped in minimizing antioxidative load, were the repercussions of enhanced gene expression in microbe inoculated plants. These mechanisms contributed strongly towards stress mitigation. The study demonstrated that microbial inoculants were successful in improving intrinsic biochemical and molecular capabilities of rice plants under stress. Results encouraged us to advocate that the practice of growing plants with microbial inoculants may find strategic place in raising crops under abiotic stressed environments.

## Introduction

Under normal environmental conditions, crop plants maintain a delicate balance in optimum growth, development and productivity. However, under nutrient limiting conditions or environmental stresses, plants face physiological and biochemical challenges leading to growth disruption due to disturbed primary metabolism^1^. Cells further suffer from oxidative damage due to the accumulation of reactive oxygen species (ROS) (superoxide, OH^−^ radical, H_2_O_2_ and singlet oxygen)^2^. ROS generated during aerobic metabolic processes usually impact cellular targets in concentration-dependent manner^3^. Normal ROS concentration in the cells regulates key cellular physiology and redox-sensitive metabolic mechanisms. However, increased level of ROS in plants growing under oxidative stresses becomes cytotoxic^4^. When exposed to abiotic stresses, normal pathways for photorespiration, photosynthesis and mitochondrial respiration lead to produce excessive ROS^5^ that disturbs intrinsic cellular homeostasis^6^. Environmental stresses also trigger activity of monoamine oxidase (MAO), xanthine oxidase (XOD) and NADPH oxidase that balance production and accumulation of ROS^7^. The consequences are observed in terms of negative cellular metabolic functions that damage nucleic acid, protein, lipid and carbohydrate metabolism^2^.

Plants are evolved with a sophisticated system to overcome ROS burden within the cells through prominent antioxidative defense mechanisms^8^. Enzymatic antioxidative mechanisms include regulation of the enzymes like superoxide dismutase, catalase, peroxidase, glutathione reductase, glutathione S-transferase and guaiacol peroxidase. These enzymes prevent or repair the oxidative damage caused due to disrupted cellular homoeostasis under stress conditions^5^. Cells also synthesize diverse antioxidant molecules that regulate signal pathways in redox mechanisms to overcome oxidative damage^4^. Increased production of antioxidative enzymes like SOD, POD, CAT, GPX and GST^9^ and the accumulation of antioxidant compounds such as carotenoids^10^ and phenylpropanoids^11^ successfully help plants reduce their load of ROS within the cells. These processes cumulatively help plants mitigate burden of oxidative mechanisms while maintaining their growth and development under stressful conditions.

Among various devastating environmental stresses for plants, drought conditions, either moderate to intense or short to prolonged, have remained a challenge for crop productivity^12^. Drought adaptation, avoidance and/or mitigation strategies in crop plants lie with their intrinsic metabolic and molecular mechanisms which, when triggered by environmental stimulus strengthen plant growth, development and productivity^13^. Beneficial microbial interactions with plants either under normal growth conditions or in stressful environment manifest diverse physiological, biochemical and molecular roles^14–16^. Microbial communities, the most natural inhabitants of the soils and the rhizosphere, the specific ecological niche associated with the root vicinity, tremendously influence plant growth and productivity^17,18^. Their interaction with the plant root system constitutes the most complex and intricate biological phenomenon that helps plant activate their adaptive capabilities against drought stress through induced defense mechanisms^19,20^. Plant growth promoting rhizobacteria (PGPR) colonize rhizosphere to promote growth and induce systemic drought tolerance^21,22^ through phytohormone, epoxypolysaccharides and ACC deaminase production^23–26^. Plant responses to *Trichoderma* inoculation as a biocontrol agent are manifested by early escape of abiotic stresses through activation of antioxidant machinery^27,28^. Inoculation of *T. harzianum* helped plants alleviate water deficit in tomato^29^ and rice^28^ through enhanced activation of ascorbate and glutathione-related defense enzymes^30^. Cumulatively, microbe-plant interaction and the resultant metabolic changes are being realized as a real time stress tolerance strategy in the plants for their survival and sustainable productivity^31^.

Rice (*Oryza sativa* L.) is the most important crop that feeds almost half of the world’s population^32^. Being a crop of tropical and subtropical origin, rice is usually sensitive to abiotic stresses, especially to drought conditions^33^. Water deficit is amongst the major limiting factors to produce rice in many parts of the world^34^. High sensitivity to drought and water deficit poses serious threat towards enhanced productivity of this crop^35^. Microbial communities are the dominant natural inhabitants of the plant rhizosphere^36,37^ including rice crop^38,39^. Their colonization and interaction with the rice roots impart beneficial plant growth promotion and abiotic stress mitigation impacts^40,41^. We demonstrated that the individual and combined inoculation of rice with *Pseudomonas fluorescens* and *Trichoderma asperellum* (T42) have contributed to strengthen intrinsic mechanisms in rice plants, thereby offering protective support against drought. Enzymatic and non-enzymatic antioxidant reactions in plants grown with microbial inoculation under non-drought and drought conditions were improved. The expression of defence-related genes that helped plants regulate ROS as key steps in microbe-mediated stress mitigation processes was explored. The study reveals that growing plants under microbe-inoculated conditions leads to modulate intrinsic biochemical and molecular mechanisms to help plants mitigate drought conditions. The observations warrant microbial inoculation as an efficient stress mitigation strategy for rice crop challenged with drought stress in the fields.

## Materials and Methods

### Seeds, microbial inoculants and experimental conditions

Seeds of rice variety Pusa Basmati (PB) 1612 were obtained from the seed bank of ICAR-Indian Institute of Seed Science, Mau, India. Rhizosphere compatible bioagents namely *Pseudomonas fluorescens* (*Pf*) (OKC; Genbank accession No. JN128891) and *Trichoderma asperellum* (*Th*) (T42; GenBank accession No. JN128894) were obtained from the Department of Mycology and Plant Pathology, Institute of Agricultural Sciences, Banaras Hindu University, Varanasi, India. Rice seeds were treated with both the cultures as described by Patel *et al*.^42^. For seed treatment, spore suspension of *Th* (spore count 1.3 × 10^8^ ml^−1^) and cell suspension of *Pf* (1.2 × 10^8^ cells ml^−1^, optical density equivalent to 0.39) was prepared in 0.5% sterilized carboxymethylcellulose (CMC). For combined application, equal proportion of fungal spores and bacterial cell suspension was mixed together and applied. Rice seeds (variety PB 1612) were surface sterilized with 0.1% HgCl_2_ solution for 2 min followed by washing thrice with sterilized distilled water. Dried seeds were coated with the inoculant suspension (individual and in combination) and kept for 3 h in air under sterilized conditions. Microbe-coated seeds were sown in earthen pots (10 inch diameter) containing 5.4 kg sterilized soil mixed with 20% vermicompost. Pots were kept in well-ventilated glasshouse throughout the Kharif season of 2017 from mid-June to November. Temperature ranged from 16.4 to 31.5 (min) to 30.1 to 39.2 °C (max) with gradual decrease as the plant development approached maturity. Regular watering was applied prior to flowering stage, before the onset of which, 7 days of continuous drought was given to one set of pots sown with the microbe-inoculated and non-inoculated (NI) rice seeds. All the plants were harvested after completion of drought period and leaves were collected for further experimentation.

### Physicochemical determination

#### Plant growth parameters

Along with the protein concentration, dry shoot and root weight were recorded under inoculated, non-inoculated, stressed and non-stressed conditions. Protein concentration was estimated following Lowry *et al*. method^43^ in which bovine serum albumin (BSA) was used as standard. Protein concentration was expressed as mg protein per gram fresh wt. Plant shoot and root wt were recorded on dry wt basis by randomly uprooting 4 plants from 6 pots, each of which contained 2 plants. Shoot and roots were dried in an oven at 65 ± 2 °C for 72 h, the total dry matter (TDM) of shoot and root was weighed separately and recorded as g per plant.

#### Quantification of H_2_O_2_

Leaf samples (0.1 g) from each treatment were homogenized in 2.0 ml 0.1% (w/v) trichloroacetic acid (TCA) and centrifuged (12,000 *g*, 15 min). The supernatant (0.5 ml) was added with 10 mM phosphate buffer (pH 7.0). Afterwards, potassium iodide solution (1 M, 1 ml) was added following incubation for 5 min. The oxidation product formed was examined at 390 nm^44^. The concentration of H_2_O_2_ formed was determined as nMol H_2_O_2_ g^−1^ fresh weight (FW).

#### *In situ* examination of cell death

*In situ* cell death determination was carried out by treating plant leaves with 0.1% Evans blue solution. After 15 min, leaves were dipped in 95% boiling ethanol (30 min) for depigmentation. Necrotic spots were identified as indigo blue lesions at the leaf surface^45^.

#### Determination of total polyphenolic content (TPC)

TPC was determined following the method of Zheng and Shetty^46^ with modifications. Leaf tissues (0.1 g) were macerated in 5 ml water:methanol (1:1, v/v) at 4°C and extracted for 48 h. Homogenized samples were centrifuged at 15000 *g* (10 min). Polyphenolic content was quantified using Folin–Ciocalteau reagent. The extract (1 ml) was mixed with water:methanol (1:1, 1 ml, v/v), distilled water (3 ml) and Folin–Ciocalteau regent (0.5 ml) followed by thorough mixing. The reaction mixture containing 5% sodium carbonate (1 ml) was kept for 30 min and examined at 725 nm. TPC was calculated as mg gallic acid equivalents (GAE) per g FW.

#### Quantitative determination of enzymes

One g of fresh rice leaves were washed with the sterilized distilled water and macerated with 5 ml phosphate buffer (pH 7.8) in ice cooled pestle-mortar kept at 4 °C. The extract was centrifuged at 15,000 rpm for 15 min at 4 °C and used for enzymatic assays.

#### Superoxide dismutase (SOD)

SOD (EC 1.15.1.1) activity was determined by photochemical reduction method of nitrobluetetrazolium (NBT) chloride^47^. Reaction mixture containing methionine (200 mmol l^−1^), NBT (2.25 mmol l^−1^), EDTA (3 mmol l^−1^), phosphate buffer (100 mmol l^−1^; pH 7.8) and sodium carbonate (1.5 mol l^−1^) was mixed with the enzyme extract. In 3 ml final volume, 2µmol l^−1^ riboflavin (0.4 ml) was added following exposure to light (15 W fluorescent lamp, 15 min). The absorbance was taken at 560 nm after deactivating the enzyme activity in dark. One unit of SOD decreased the absorbance by 50% as compared to control, which lacked enzyme extract.

#### Peroxidase (PO)

PO (EC 1.11.1.7) was estimated in the reaction mixture containing 1.5 ml pyrogallol (0.05 mol), 0.05 ml enzyme extract and 0.5 ml H_2_O_2_ (1%; v⁄v)^48^. The change at 420 nm was determined at every 30 s intervals and the enzyme activity was recorded as U per min per g FW.

#### Ascorbate peroxidase (APX)

Plant leaves (100 mg) were suspended in 0.1 M sodium phosphate buffer (pH 6.8) containing 2 mM ascorbate, homogenized and centrifuged (15000 *g*, 20 min). The reaction mixture containing phosphate buffer (25 mM, pH 7.0), EDTA (0.1 mM), ascorbic acid (0.25 mM), H_2_O_2_ (1.0 mM) and enzyme extract (0.2 ml) was kept at room temp^49^. Reduction in absorbance was measured at 290 nm after 60 s and activity was expressed as U min^−1^ g^−1^ FW.

#### Catalase (CAT)

CAT (E.C. 1.11.1.6) was assayed by Aebi method^50^. Reaction mixture consisting of phosphate buffer (300 µM, pH 7.2) and H_2_O_2_ (100 µM) in enzyme extract (1 ml) was allowed to release O_2_ by enzymatic dissociation of H_2_O_2_ in the dark for 1 min. O_2_ produced due to enzyme reaction was determined at 240 nm (extinction coefficient of H_2_O_2_ is 0.036 mM^−1^ cm^−1^). The activity of the enzyme was expressed as µM H_2_O_2_ oxidized U min^−1^ g^−1^ FW.

#### Glutathione reductase (GR)

The method of Anderson^51^ was followed to determine GR (E.C. 1.6.4.2) activity. Reaction mixture contained Tris–HCl buffer (50 mM, pH 7.6), NADPH (0.15 mM, 10 ml), oxidized glutathione (1 mM GSSG, 100 µl), MgCl_2_ (3 mM) and enzyme extract (0.3 ml). GR was measured as gradual reduction in absorbance of NADPH at 340 nm. The activity of the enzyme was calculated in terms of U (nmol oxidized NADPH) min^−1^ mg^−1^ FW.

#### Guaiacol peroxidase (GPX)

GPX (E.C. 1.11.1.7) was measured by recording the increase in absorbance at 470 nm^52^. The reaction mixture consisting of sodium phosphate (10 mM; pH 6.0), H_2_O_2_ (0.3%, v/v), tetraguaiacol (1%, v/v) and enzyme extract (0.3 ml) was prepared. The enzyme activity was represented in terms of U min^−1^ mg^−1^ FW where one unit of enzyme catalyzes the oxidation of 1µmol of guaiacol min^−1^.

#### Phenylalanineammonia lyase (PAL)

Powdered leaf samples (0.5 g) were homogenized in 5 ml of ice-cold phosphate buffer (100 mM; pH 7.0 and 0.5 mM EDTA and mixed with 1.4 mmol l^−1^ β-mercaptoethanol^53^. The homogenate was centrifuged (15000 *g*, 15 min) and the supernatant was added with 0.1 mol l^−1^ l-phenylalanine (pH 8.7, 1 ml) along with the mixture of 0.5 ml 0.2 mol l^−1^ phosphate buffer (pH 8.7), 0.2 ml enzyme extract and 1.3 ml distilled water following incubation for 30 min. Trichloroacetic acid (TCA, 0.5 ml, 1 mol l^−1^) was added to terminate the reaction. The observations were recorded at 290 nm and activity was expressed in terms of µmol t-cinnamic acid g^−1^ FW.

### Estimation of non-enzymatic antioxidative reactions

#### Free radical scavenging activity (FRSA)

The free radical scavenging activity was evaluated by 2,2-diphenyl-1-picrylhydrazyl (DPPH) radical scavenging method using the stable radical DPPH^54^. Plant extract with TPC (100 μl) was mixed with 2.9 ml freshly prepared DPPH solution (60 μM in MeOH). The reduction in DPPH radical was determined at 515 nm till 1 h until stable values were obtained.

#### ABTS activity

The ABTS activity in TPC from the rice leaf was determined using the ABTS• + decolorization method^55^. The reaction mixture containing 10 ml ABTS• + radical (ABTS 9.5 mL, 7 mM) and potassium persulfate (245 μL, 100 mM) was kept in the dark for 18 h and then diluted with potassium phosphate buffer (0.1 M, pH 7.4) to an absorbance of 0.70 (±0.02) at 734 nm. TPC from rice leaves (50 μL) was mixed thoroughly with 2.95 mL ABTS radical solution. The absorbance was recorded at 734 nm and expressed as % inhibition of the activity.

#### Ferric reducing power antioxidant assay

The Fe-ion reducing power assay was performed with the leaf extracts taking quercetin as the standard compound^56^. To 200 and 500 μl aliquots, 1.0 ml MeOH, 2.5 ml phosphate buffer (pH 6.6) and 1% (w/v) potassium ferricyanide were added. Reaction mixture was incubated at 50 °C for 20 min and 2.5 ml TCA (10% w/v) was added to terminate the reaction. Absorbance was recorded at 700 nm and percent increase in Fe reducing activity was calculated.

#### Ferrous ion chelation activity

Ferrous ion chelation was determined by the method of Decker and Welch^57^. Rice leaf extract (200 μl) was maintained to 1.0 ml with MeOH and then added with 0.1 ml of ferrous chloride (2.0 mM), ferrozine [3-(2-pyridyl)-5,6-bis-(4-phenylsulfonic acid)-1,2,4-triazine] (0.2 ml of 5.0 mM) and methanol (3.7 ml). After 10 min, the absorbance was recorded at 562 nm where low absorbance indicated high ferrous ion chelating capacity.

#### Total RNA isolation and cDNA synthesis

Total RNA was isolated from 0.2 g fresh rice leaves using TRIzol™ LS reagent (Invitrogen; http://www.invitrogen.com). Three µg of the total RNA was digested with RNase-free DNase I (Thermo Scientific) to remove genomic DNA contamination. The poly(A)-RNA concentration was determined using NanoDrop 2000 spectrophotometer (Thermo Scientific). Samples with a 260/280 ratio of 1.9–2.1 and a 260/230 ratio ≥ 2.0 were chosen to determine the quality and purity of the RNA preparations. The integrity of the purified RNA was checked on 2% formamide denaturing gel. Subsequently, first-strand cDNA was synthesized in a 20 μL reaction mixture by using RevertAid H minus kit (Fermentas) following the manufacturer’s instructions and stored at −20 °C until use.

#### Quantitative qRT-PCR assay

Gene specific primer sequences for the defense-related genes as listed in Table 1 were obtained from TIGR Rice Genome Annotation Resource^58^ with the help of BLASTn and were synthesized from Helix Biosciences, India. qRT-PCR amplification was performed in 96-well plates with a iQ5 RT-PCR Detection System (BioRad Laboratories, Germany) using Green Supermix Kit Eva Green SYBR^®^ (BioRad). Expression of the gene specific primers at a concentration of 0.1 µM was analyzed^42^. In brief the qPCR conditions were: denaturation at 95 °C for 2 min followed by 40 repeats at 95, 60 and 72 °C temp for 20, 30 and 25 s. The sense/antisense primer sequences for actin (5'-TCCATCTTGGCATCTCTCAG-3'/5'-GTACCCTCATCAGGCATCTG-3') and rRNA (5'- CTTCGGGATCGGAGTAATGA-3'/5'-AACTAAGAACGGCCATGCAC-3'), respectively were used as internal controls for normalizing relative gene expression levels in technically independent and triplicate biological experiments^59^. The threshold cycle (Ct) was measured automatically by the software.

**Table 1:** Gene specific forward (F) and reverse (R) primers used in the study.

Sl. No.	Gene Name		Primer 5′-3′
1	OsPIP1;1	F	TACATGGGCAATGGCGGT
		R	CAAGACCGTCACCCTTGGTG
2	DHN LOC_Os01g50700	F	CAGCTCCAGCTCGGTAACTT
		R	CTTCTGCTCCTCCTGCTTGT
3	DREB LOC_Os09g35030	F	GGAGCAAGCAGAAACACACA
		R	TCGTCTCCCTGAACTTGGTC
4	cCuZn-SOD1	F	GAGATTCCAAACCAGCAGGA
		R	TTGTAGTGTGGCCCAGTTGA
5	Fe-SOD	F	CTTGATGCCCTGGAACCTTA
		R	GCCAGACCCCAAAAGTGATA
6	Mn-SOD1	F	GGAGGCCATGTCAATCATTC
		R	CACAAGGTCCAGAAGTGCAA
7	Chl_sAPX	F	CAATTGAGGAAGCTGGTGGT
		R	ACTTCAGCGATCTGGCTCAT
8	CATa	F	CCACCACAACAACCACTACG
		R	CCAACGACTCATCACACTGG
9	AU076282	F	GCTACTACCGCAACCTCGTC
		R	TCACTTTCCTGCAGTTGAGC
10	D14481	F	CGCGATAAAGGAAGATCTCG
		R	CGTCATAGTAAGGGCCTCCA
11	PAL 1	F	CAGACACGGTCGTACCATTG
		R	CCACCTCCTGCATTTGTTTT

#### Statistical analysis

Data were subjected to Two Way ANOVA using PRISM version 5.0. Tests for normality of data and for homogeneity of variances were performed before running ANOVAs. PCA analysis was carried out using R-program. Except for the real time experiments using qRT-PCR, for which three replications were used, all the experiments were performed in complete randomized block design having six replications (n = 6). For the gene expression analyses, the expression values of the two housekeeping genes (actin and rRNA) were subjected to Two-way ANOVA using geometrical means of the internal controls and based on the mean values, the expression profile of all the genes was normalized. For all the experiments, the data were expressed as the mean value of the replicates. Standard error for each mean value was represented separately in the table and figures.

## Results and Discussion

Plant responses to abiotic stresses are growth dependent and complex^60^. The underlying array of mechanisms for stress avoidance, tolerance and adaptation are conditional constraints involving multiple cellular physiological, metabolic and molecular alterations^31^. Stress induced antioxidative conditions within the cells generate reactive oxygen species (ROS) and lead to accumulation of free radicals that disrupt cellular homeostasis and adversely affect cell viability^61^. Stressed plants undergo multiple intrinsic equilibrations for early stress perception, signal channeling, gene expression and metabolic modifications to refrain from unfavorable conditions^62^. Microbial interactions with plants elicit modulation in molecular mechanisms to activate metabolic networks at gene, enzyme and metabolite level. This works in parallel to enhance plant’s intrinsic strength to support stress mitigation^63^. We inoculated rice with the strains of *Trichoderma* (*Th*) and *Pseudomonas* (*Pf*) as individual and combined inoculants (*Th* + *Pf*) and assessed whether microbial inoculation helped plants improve their metabolic capabilities to combat drought and if so, to what extent the biochemical and molecular level changes were linked with stress mitigation.

### Microbial inoculation supports plant growth under drought stress

Protein concentration is one of the most prominent parameters to assess the impact of microbial inoculation on plants grown under drought or non-drought conditions. As indicated by two way ANOVA, the main effects of watering regime [F(1,40) = 281.8, *p* < 0.0001] and microbial inoculation [F(3,40) = 145.5, *p* < 0.0001] on protein concentration was significant. The impact of interaction of drought and microbial inoculation was also statistically significant [F(3,40) = 18.06, *p* < 0.0001]. In non-inoculated control plants, the concentration of protein (mg g^−1^) was significantly different (M = 8.96, SD = 0.51) than in the plants challenged with drought (M = 12.51, SD = 0.51). Maximum protein concentration was observed in plants challenged with drought and inoculated with *Th* + *Pf* (M = 16.90, SD = 0.5) followed by those inoculated with *Th* (M = 15.50, SD = 0.55) and Pf (M = 13.50, SD = 0.56) and grown under drought condition. Pair-wise analysis indicated significant differences between control and *Th*, *Pf* and *Th* + *Pf* inoculated non-drought plants. Further, the protein concentration in control plants was also significantly different than those grown under drought condition or in the plants challenged with the drought and given microbial inoculation (Table 2). Drought or desiccation tolerance in plants is known to promote accumulation of biomolecules including proteins^64^.

**Table 2:** Impact of microbial inoculation on protein concentration and shoot and root dry weight of rice plants grown under non-drought and drought-challenged conditions.

Treatments Parameters	Control (Non-Inoculation)	*Trichoderma* inoculation (*Th*)	*Pseudomonas* inoculation (*Pf*)	Combined inoculation of *Trichoderma* & *Pseudomonas (Th* + *Pf*)	Statistics				
Protein Concentration (mg g^−1^)					Source	*df*	MS	*F*	*P*
Non-Drought	8.96 ± 0.51^d^	12.91 ± 0.32^c^	12.74 ± 0.41^c^	13.30 ± 0.80^c^	Drought	1	82.58	281.8	<**0.0001**
					Inoculation	3	42.64	145.5	<**0.0001**
Drought	12.51 ± 0.51^c^	15.50 ± 0.55^b^	13.50 ± 0.56^c^	16.90 ± 0.50^a^	Drought*Inoculation	3	5.29	18.06	<**0.0001**
					Error	40	0.2898		
**Shoot dry weight (g per plant)**									
Non-Drought	5.48 ± 0.39^cd^	6.66 ± 0.83^bc^	7.78 ± 0.34^ab^	8.21 ± 1.11^a^	Drought	1	6.42	10.85	**0.0021**
					Inoculation	3	16.92	28.58	<**0.0001**
Drought	4.81 ± 0.82^d^	6.09 ± 1.06^cd^	6.75 ± 0.59^bc^	7.56 ± 0.58^ab^	Drought*Inoculation	3	0.1215	0.205	0.892
					Error	40	0.5899		
**Root dry weight (g per plant)**									
Non-Drought	5.84 ± 0.21^ef^	6.86 ± 0.77^cd^	7.77 ± 0.65^bc^	8.93 ± 0.40^a^	Drought	1	4.23	16.67	**0.0002**
					Inoculation	3	15.96	62.89	<**0.0001**
Drought	5.51 ± 0.44^f^	6.62 ± 0.29^de^	6.96 ± 0.38^cd^	7.93 ± 0.60^b^	Drought*Inoculation	3	0.4057	1.598	0.2049
					Error	40	0.2507		

*p* values in Bold are significantly different.

Drought reduced shoot and root dry weight although microbial inoculation substantially supported plant growth. Results of the two-way ANOVA for shoot dry weight showed significant effects of watering regime [F(1,40) = 10.85, *p* = 0.0021] and microbial inoculations [F(3,40) = 28.58, *p* < 0.0001], while the interaction effect was not significant [F (3,40) = 0.205, *p* 0.892] (Table 2). Shoot dry weight values of well-watered plants (M = 5.48, SD = 0.39) were significantly higher than those of drought-stressed plants (M = 4.81, SD = 0.82). On the other hand, *Th* + *Pf* inoculated plants had the highest shoot dry weight (M = 8.21, SD = 1.11), followed by *Pf* inoculated plants (M = 7.78, SD = 0.34), *Th* inoculated plants (M = 6.66, SD = 0.83) and uninoculated control plants (M = 5.48, SD = 0.39). Tukey’s pairwise tests indicated significant differences (*p* < 0.05) in between non-drought (control) plants and both *Pf* and *Th* + *Pf* inoculated plants, but no significant differences either between control and *Th* inoculated drought treated plants or between non-drought *Pf* and *Pf* + *Th* inoculated drought treated plants (Table 2). Similarly, on root dry wt, the impact of drought [F(1,40) = 16.67, *p* = 0.0002] and microbial inoculation [F(3,40) = 62.89, *p* < 0.0001] was statistically significant but the impact of interaction was non-significant [F(3,40) = 1.598, *p* < 0.2049]. Reduction in growth parameters in rice is the most obvious negative impact of drought and water deficit^65^. We reported that despite drought, microbial inoculation supported growth and development of shoot and root of rice plants in almost similar way as was evidenced under non-drought condition. Therefore, the negative impact of one factor (drought) is substantially being compensated by the other factor (microbial inoculation). Since growth promoting microorganisms enhance nutrient uptake by the plants, produce phytohormones and stimulate plant’s immune system^14^, the observed effect of microbial inoculation on developmental parameters, even in stress challenged plants, seems natural. These observations provided evidence that microbial inoculation may protect plants by bringing positive changes at physiological and morphological level under drought challenged condition.

### Microbial inoculants help plants tolerate H_2_O_2_ impact and hypersensitive cell death

**Figure 1:**
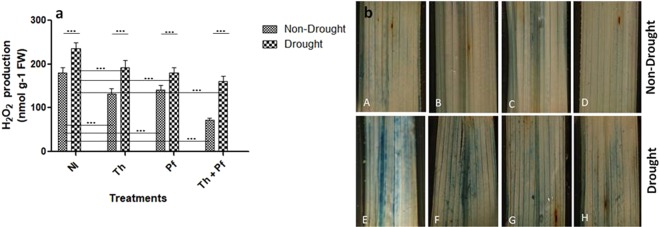
Generation of H_2_O_2_ in plants grown with microbial inoculation and post-drought stress (**a**) and *in situ* hypersensitive response in leaves (**b**). A & E: NI (non-inoculated); B & F: *Trichoderma* inoculation (*Th)*; C & G: *Pseudomonas* inoculation (*Pf*); D & H: combined inoculation (*Th* + *Pf)*. Level of significance was determined by two-way ANOVA. n = 6. Data are mean ± SEM.

H_2_O_2_ level in the rice plants was reduced due to microbial inoculation. Compared to the non-inoculated control plants with high H_2_O_2_ level [M = 179.6 nmol g^−1^ FW, SD = 13.06], non-stressed plants inoculated with *Th* showed [M = 132.93, SD = 11.95], *Pf* [M = 141, SD = 10.07] and *Th* + *Pf* [M = 71.73, SD = 5.16] H_2_O_2_ concentration (Fig. 1a). Between control and the drought plants, the values of H_2_O_2_ content were significantly different. Pairwise tests also indicated significant differences in between non-inoculated drought challenged plants with those challenged with the drought and inoculated with the microbial species. Drought showed significant impact on H_2_O_2_ level in plants [F(1,40) = 296.9; *p* < 0.0001]. Microbial inoculation to plants also showed significant effects on H_2_O_2_ concentration in plants [F(3,40) = 112.1, *p* < 0.0001]. The interaction impact of drought and microbial inoculation was again significant [F(3,40) = 8.388, *p* = 0.0002) (Fig. 1a, Supplementary Table 1). We showed that although drought led to high H_2_O_2_ level, microbial inoculation lowered the magnitude of accumulation and thereby, lowered the toxic effect of H_2_O_2_ in the cells. This is further evidenced from the *in situ* hypersensitive reaction in the leaves of the rice plants (Fig. 1b). Leaves of non-stressed plants (Fig. 1b,A) grown with microbial inoculation (Fig. 1b,B,C,D) remained almost free from the lesions. Leaves of the plants grown under drought showed maximum stained lesions (Fig. 1b,E). However, microbial inoculation helped stressed plants minimize hypersensitive spots on the leaves (Fig. 1b,F,G) and minimum lesions were seen over the leaves of the plants inoculated with *Th* + *Pf* (Fig. 1b,H). Higher accumulation of H_2_O_2_ in plant cells is a toxic phenomenon leading to hypersensitive cell death. Microbial inoculation not only reduced the level of H_2_O_2_ in drought stressed plants, but it also minimized lesion development due to hypersensitive cell death in plant leaves. Drought as an unfavorable condition leads to the overproduction of H_2_O_2_ that eventually increased phytotoxicity leading to cell necrosis. Existing reports further confirm such processes in plants experiencing stressed conditions^66–68^.

### Polyphenolics and PAL activity during drought stress

**Figure 2:**
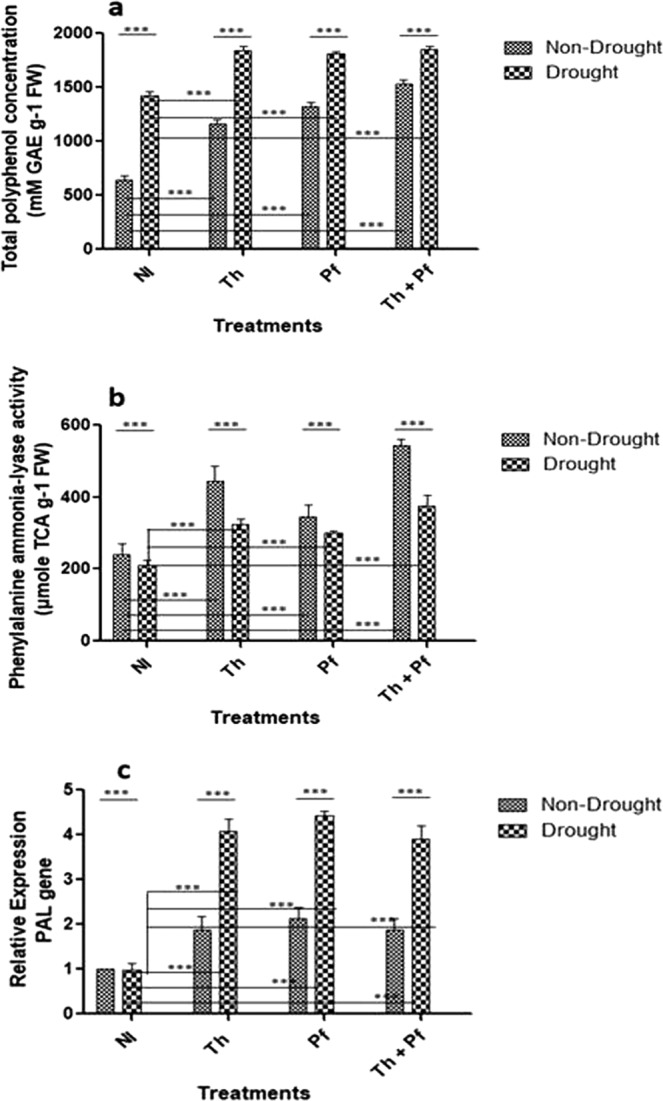
Accumulation of total polyphenol concentration (TPC) (**a**), PAL enzyme activity (**b**) and expression of *PAL* gene (**c**) in the leaves of microbe-inoculated and non-inoculated rice plants grown under non-drought and drought challenged conditions. GAE = gallic acid equivalents; TCA = trichloroacetic acid; Significance level was determined using two-way ANOVA. Data are mean ± SEM. n = 6 for TPC and enzyme assay, n = 3 for transcript analysis.

Accumulation of polyphenolics in plant leaves is shown to have protective role against stresses through anti-oxidation and ROS deactivation^69,70^. Polyphenolic metabolites play important role in plant defense against abiotic and biotic stresses^71^. Results of the two-way ANOVA for total polyphenol concentration showed significant effects of watering regime [F(1,40) = 549.2, p < 0.0001] and microbial inoculations [F(3,40) = 141.5, *p* < 0.0001]. The interaction effect was also significant [F(3,40) = 17.77, *p* < 0.0001] (Table S1). Drought-stressed plants had always significantly higher total polyphenol concentration than non-stressed plants (Fig. 2a). On the other hand, one-way ANOVAs and post hoc Tukey’s tests on both the drought stressed and non-stressed plant cohorts showed that significantly the lowest total polyphenol concentration was always in uninoculated plants. Among the three inoculation treatments in the cohort of drought-stressed plants, combined inoculation resulted in significantly high polyphenol concentration. Also, in the cohort of non-stressed plants, plants doubly inoculated with *Trichoderma* and *Pseudomonas* (*Th* + *Pf*) had significantly higher (p < 0.05) total polyphenol concentration than singly inoculated plants (Fig. 2a).

Microbial inoculation resulted in enhanced activity of PAL enzyme in rice leaves. One way ANOVA and Tuckey’s test results on drought and non-drought plants indicated that significantly low PAL activity was always reflected in stressed plants (Fig. 2b). In the cohort of non-stressed plants that always showed higher PAL activity than stressed plants, those with combined inoculation of *Th* + *Pf* had significantly high PAL activity than any other single microbial inoculation. In non-stressed plants without inoculation, PAL activity was [M = 240.77 µM TCA g^−1^ FW, SD = 30.88]. In non-stressed plants inoculated with *Th* the activity was [M = 443.2, SD = 43.38], with *Pf* it was [M = 344.1, SD = 35.11] and *Th* + *Pf* it was [M = 543.7, SD = 16.01] (Fig. 2b). The impact of watering regime on PAL activity in plants was significant [F(1,40) = 135.0, *p* < 0.0001]. The impact of microbial inoculation was also significant [F(3,40) = 163.0, *p* < 0.0001] and so was the effect of interaction [F(3,40) = 17.04, *p* < 0.0001 (Fig. 2b). Microbial inoculation to plants under stressed condition influences accumulation of polyphenolics and activates PAL enzyme activity^72–75^. Since polyphenolics are strong antioxidants and PAL is a defense-related enzyme, high accumulation of polyphenolics and enhanced PAL enzyme activity in the leaves are supposed to strengthen plants under drought challenged condition. Having shown that the microbial inoculation enhanced polyphenolic accumulation and improved PAL enzyme activity, the expression of *PAL* gene was checked in plant leaves (Fig. 2c). Microbial inoculation enhanced *PAL* gene expression in the non-drought plants. In the cohort of plants grown under drought following microbial inoculation, expression of *PAL* gene was multi-fold enhanced (Fig. 2c). The impact of watering regime on *PAL* gene expression was statistically significant [F(1,16) = 102.5, *p* < 0.0001]. The effect of microbial inoculation showed statistical significance [F(3,16) = 42.08, *p* < 0.0001]. The interaction impact also had statistically significant values [F(3,16) = 11.79, *p*0.0003] (Supplementary Table 2). Stressed conditions usually activate phenylpropanoid pathway, in which *PAL* is a key gene to offer physiological and structural support to the plants^76,77^. A correlative activation pattern of the *PAL* gene, the enzyme activity and accumulation of polyphenolics in the leaves of rice plants grown with microbial inoculation was found under drought stress. Such biochemical and molecular strategies are presumed to confer cumulative support to rice to tolerate the adverse impact of stress.

### Polyphenolics accumulation enhanced antioxidant profile in inoculated plants

Normal concentration of intracellular ROS regulates redox state in the cells and also acts as signals for defense against stresses^78,79^. Unfavourable conditions enhance production and prolonged accumulation of ROS in cellular compartments^80^, a condition that becomes phytotoxic with deleterious impact due to oxidative damage of cell membrane^81,82^. Small molecule metabolites like phenolics, tocopherol, carotenoids and proline maintain redox state in cells during oxidative damage as ROS scavengers^2,83^. This is why enhanced polyphenolics concentration usually favours ROS scavenging in the plants grown under stress conditions.

**Figure 3:**
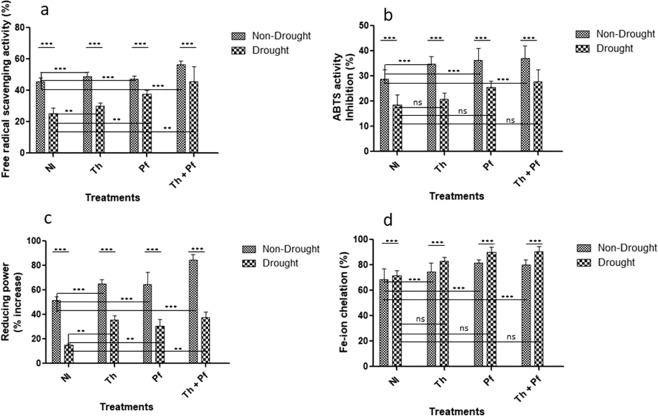
Impact of microbial inoculation on the antioxidant activity of the leaf extract of rice plants grown under non-drought and drought challenged condition. Free radical scavenging activity (**a**), ABTS activity (**b**), Reducing power assay (**c**) and Fe-iron chelation activity (**d**). The level of statistical significance was determined by two-way ANOVA; ns is non-statistical significance; n = 6; Data are mean values ± SEM; ns is non-significant.

Two way ANOVA results showed that the effect of microbial inoculation on free radical scavenging activity (FRSA) of polyphenolic extract from rice plants had significant values [F(3,40) = 29.85, *p* < 0.0001] (Fig. 3a; Supplementary Table 3). Within the group of plants grown under non-stressed condition, the plants receiving combined inoculation of *Th* + *Pf* showed high FRSA activity [M = 56.29, SD = 2.66] as compared to control plants [M = 45.58, SD = 2.46] and single inoculations. The impact of drought on FRSA activity was significant [F(1,40) = 151.7, *p* < 0.0001], so was the impact of interaction [F(3,40) = 5.154, *p*0.0042]. Reduction in the radical cation as measured through decolorization of ABTS^•+^ was observed. Polyphenolic extract of rice leaves from the cohort of non-stressed plants inoculated with microbial inoculants showed high ABTS inhibition in comparison to stressed and microbe inoculated plants. Doubly inoculated plants showed high inhibition of ABTS activity [M = 37.08, SD = 5.05] than control (M = 28.79, SD = 3.84) and single *Th* [M = 34.86, SD = 2.98] or *Pf* [M = 36.31, SD = 4.65] inoculation (Fig. 3b). Two way ANOVA results showed that the effects of microbial inoculation on ABTS inhibition was significant [F(3,40) = 11.80, *p* < 0.0001]. The impact of watering regime was again found to be significant [F(1,40) = 96.15, *p* < 0.0001]. However, the impact of interaction of inoculation *vs* drought was statistically non-significant [F(3,40) = 0.8662, *p*0.4666]. Reduction of Fe^3+^-ferricyanide complex to Fe^2+^ by the plant extracts is an important assay to measure antioxidant activity in terms of reducing power (RP). Reduced RP activity was observed in the cohort of plants challenged with drought and inoculated with microbial species. Two way ANOVA results reflected that the impact of drought [F (1,40) = 639.8] and microbial inoculation [F ratio (3,40) = 61.13] was statistically significant at *p* < 0.0001 (Fig. 3c, Supplementary Table 3). The effect of interaction was also found significant [F(3,40) = 6.339, *p* 0.0013]. Within the set of non-stressed plants, doubly inoculation increased reducing power [M = 84.64, SD = 4.64] compared to single inoculation of *Th* [M = 64.94, SD = 3.69] and *Ps* [M = 64.74, SD = 9.78] and non-inoculated control [M = 51.81, SD = 2.81] (Fig. 3c).

The impact of microbial inoculation on Fe^+2^ chelation activity in plants was statistically significant [F(3,40) = 26.28, *p* < 0.0001]. The effects of drought was again found to be significant [F(1,40) = 27.63, *p* < 0.0001] (Fig. 3d, Supplementary Table 3). However, the impact of interaction was statistically non-significant [F(3,40) = 1.255, *p*0.3029]. Drought induced H_2_O_2_ production in plants has been obvious from the results (Fig. 1a) that could lead to high ROS accumulation. We presume that due to high concentration of polyphenolics in leaf extracts, rice plants show ROS scavenging strategy to neutralize the impact of oxidative toxicity. The results apparently describe that polyphenols in leaves of rice plants grown under microbial inoculation has profound non-enzymatic ROS scavenging impact. This strategy appears to be a promising stress tolerance mechanism in plants grown under drought^6,9,84^.

### Microbial inoculation activate antioxidant defense enzymes in rice

Among the antioxidant machinery against oxidative damage, plants activate antioxidant enzymes as primary ROS scavengers. Antioxidative enzymes are ubiquitous in plant cells^85^ to perform defense related action under induced oxidative stress conditions^86^. We examined elicitation of SOD, PO, APX, catalase, GR and GPX as key inducible enzymes in drought stressed plants subsequently inoculated with microbial species.

**Figure 4:**
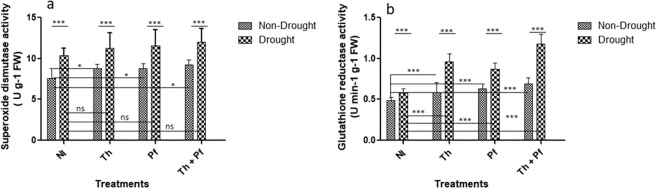
Microbial inoculation leads to enhance superoxide dismutase (SOD) (**a**) and glutathione reductase (GR) (**b**) enzyme activity in rice leaves. n = 6; Data are mean ± SEM; The level of statistical significance was determined by two-way ANOVA; ns is non-significant.

SOD with strong ROS scavenging functions catalyzes superoxide (O_2_^−^) in to O_2_ and H_2_O_2_^87^. The group of enzyme copper-zinc-SOD (Cu/Zn-SOD), iron-SOD (Fe-SOD) and manganese-SOD (Mn-SOD) is compartmentalized into the cells to act against oxidative damage^88^. SOD helps in removing O^−^_2_ from the cells by forming O_2_ and H_2_O_2_ through dismutation^89^. Enhanced activity of the enzyme so as to discard as much O^−^_2_ formed due to oxidative condition as possible is a positive sign for cellular protection^90^. In the cohort of plants grown under non-drought and drought conditions separately, microbial inoculation enhanced SOD activity. Among the treatments, doubly inoculated plants showed high values of SOD activity under both the drought and non-drought plants than single inoculation (Fig. 4a). In non-drought plants with *Th* + *Pf* inoculation, the SOD activity was high [M = 9.2, SD = 0.57] than in *Trichoderma* [M = 8.8, SD = 0.73], *Pseudomonas* [M = 8.7, SD = 0.61] inoculated and non-inoculated rice leaves [M = 7.5, SD = 1.21]. The impact of drought on the SOD activity was statistically significant [F(1,40) = 52.30, *p* < 0.0001] (Fig. 4a, Supplementary Table 4). The effects of microbial inoculation was also significant [F(3,40) = 3.598, *p*0.0216]. The interaction impact was however, found to be non-significant [F(3,40) = 0.0541, *p*0.9832] (Supplementary Table 4). Results indicated that microbial inoculation to plants enhanced SOD activity even under drought challenged conditions. It is presumed that SOD is helpful in extending the first line of defense to the plants as they play vital role as ROS scavengers.

Glutathione reductase (GR) is a potential enzyme in the antioxidative enzyme system of the plants. Two way ANOVA indicated that the effects of watering regime on GR activity in plants was significant [F(1,40) = 147.2, *p* < 0.0001] and so was the impact of microbial inoculation [F(3,40) = 44.07, *p* < 0.0001] and that of interaction [F(3,40) = 12.46, *p* < 0.0001) (Fig. 4b, Supplementary Table 4). In the cohort of plants challenged with drought and inoculated with the microbial inoculants, the value of GR activity was high in doubly inoculated plants [M = 1.18, SD = 0.12] than in plants with single inoculation of *Th* [M = 0.96, SD = 0.09] and *Pf* [M = 0.87, SD = 0.07]. Results indicated that microbial inoculation enhanced GR activity in drought challenged plants than in non-inoculated plants grown under drought. Glutathione, a tripeptide is abundant in cellular components and is widely involved in cell growth and regulation of gene expression linked with stress responses^91^. The enzyme replenishes cellular pool of glutathione that has a reductant role against detrimental ROS^83^. Multifold increase in GR activity in rice leaves following microbial inoculation, even under drought stress indicated for a defense support to the plants under microbial inoculation.

**Figure 5:**
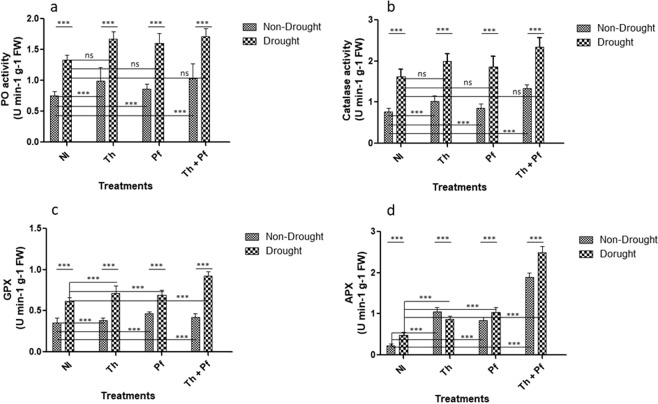
Impact of microbial inoculation and drought condition on peroxidase (**a**), catalase (**b**), guaiacol peroxidase (**c**) and ascorbate peroxidase (**d**) enzyme activity in rice plants. Significance level was determined by two-way ANOVA; n = 6; Data are mean ± SEM; ns is non-significant.

The enzyme activity of peroxidase (PO) indicates tolerance in plants against water stress^92^. We demonstrated that in the cohort of non-drought plants, microbial inoculation led to enhance peroxidase activity in rice leaves and maximum activity was found due to doubly inoculation of *Th* + *Pf*. Within the cohort of inoculated plants challenged with the drought, again doubly inoculation of *Th* + *Pf* showed maximum PO activity than single inoculation or drought plants alone (Fig. 5a). The effects of drought on PO activity was found to be significant [F(3,40) = 239.6, p < 0.0001]. The effects of microbial inoculation was also significant [F(3,40) = 11.96, *p* < 0.0001] but that of interaction was non-significant [F(3,40) = 0.6073, *p*0.6141] (Fig. 5a; Supplementary Table 5).

Catalase possesses high affinity for H_2_O_2_ and catalyzes its dismutation into H_2_O and O_2_^7,92^. In the cohort of stressed plants, plants doubly inoculated with *Th* + *Pf* had high catalase activity than single inoculations. Likewise, within the cohorts of inoculated non-stressed plants, double inoculation again led to high catalase activity (Fig. 5b). The impact of watering regime on catalase activity was significant [F(1,40) = 379.9, *p* < 0.0001] and so was the significant impact of microbial inoculation in plants [F(3,140) = 30.42, *p* < 0.0001]. However, the impact of interaction on catalase activity was found to be non-significant [F(3,40) = 0.6272, *p*0.6017] (Fig. 5b, Supplementary Table 5).

GPX reduces the level of H_2_O_2_ in the cells during stress conditions^93,94^. We showed that in the cohort of drought stressed plants, those inoculated with *Th* + *Pf* showed high GPX activity than those with single microbial inoculations (Fig. 5c). The impact of watering regime on GPX activity was significant [F(1,40)) = 423.7, *p* < 0.0001]. Similarly, microbial inoculation further showed significant effects on GPX activity [F(3,40) = 23.26, *p* < 0.0001] and so was the impact of interaction [F(3,40) = 15.40, *p* < 0.0001] (Supplementary Table 5).

The enzyme ascorbate peroxidase (APX) having great affinity for H_2_O_2_ reduces hydrogen peroxide to water molecules in chloroplasts, cytosol and mitochondria^92^. Under drought stress conditions, plants inoculated with *Th* + *Pf* showed maximum APX activity [M = 2.48, SD = 0.14] than the single inoculation of *Th* [M = 0.87, SD = 0.06] and *Pf* [M = 1.03, SD = 0.11] (Fig. 5d). The impact of inoculation of the inoculants on APX activity was statistically significant [F(3,40) = 776.9, *p* < 0.0001] in two way ANOVA. The effects of drought on APX activity was also significant [F(1,40) = 59.45, *p* < 0.0001] and so was the interaction impact on APX [F(3,40) = 33.47, *p* < 0.0001] (Supplementary Table 5).

Enhanced level of defense related enzymes is directly related to the degree of drought experienced by the plants^95^. Cell-bound peroxidases act as detoxifier of H_2_O_2_ produced as a byproduct of antioxidative mechanism^9^. The PO acts in H_2_O_2_-scaveginging and oxidize flavonoid and phenylpropanoids^72^. APX also performs H_2_O_2_ scavenging in the cytosol and chloroplast with the help of ascorbate as specific electron donor^91^. Thus, higher activity of both PO and APX is presumed to have a role in detoxification of enhanced H_2_O_2_ accumulation in the cells. Enhanced level of GPX and catalase is supposed to support plant’s biochemical strategy to mitigate drought under microbial inoculation. The enhanced activity of PO, APX, GPX and CAT enzymes in different cohorts of experiments led us to affirm the role of i) the enzyme activation and activity in imparting protection against stresses and ii) the microbial species in modulating enzyme activity in plants challenged with drought. Enhanced level of the defense related enzymes due to microbial inoculation go in parallel to different molecular mechanisms and strengthen the plant’s performance under stressed conditions.

**Figure 6:**
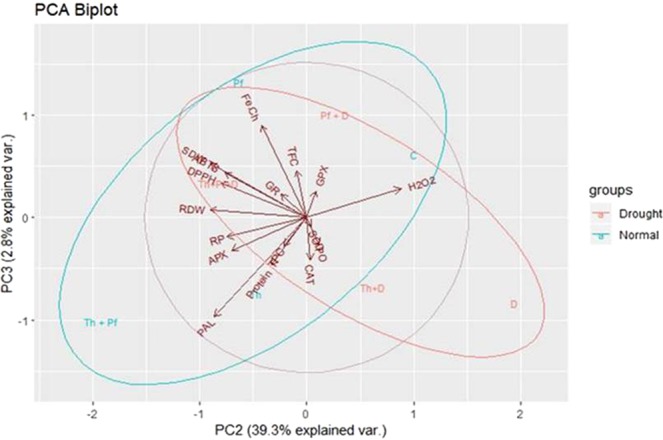
PCA analysis of enzymatic assays and plant biomass.

PCA analysis showed the effect of drought (red colored) and non-drought (normal) (indigo colored) plants in two subgroups. The effect of drought was significant on CAT, PO, SOD, GR, GPX. The quantitative level of these biochemical products was found enhanced in inoculated plants growing under drought condition than in normal irrigated plants. Total polyphenolics concentration (TPC), protein, H_2_O_2_, Fe-Chelation, ABTS and DPPH were also high in drought challenged plants than in normal irrigated plants. Apart from PAL activity, APX and RP were high in normal irrigated plants than drought challenged plants. Co-inoculation of *Trichoderma* and *Pseudomonas* improved activity of PAL, APX and RP. The activity of CAT, SOD and PO were enhanced in *Trichoderma* inoculated drought challenged plants. The other antioxidant tests such as DPPH, ABTS, GR, and iron-chelation activity were high in co-inoculation of *Trichoderma* and *Pseudomonas* inoculated and drought challenged plants (Fig. 6).

### Microbial inoculation up-regulates the genes encoding dehydration tolerance

**Figure 7:**
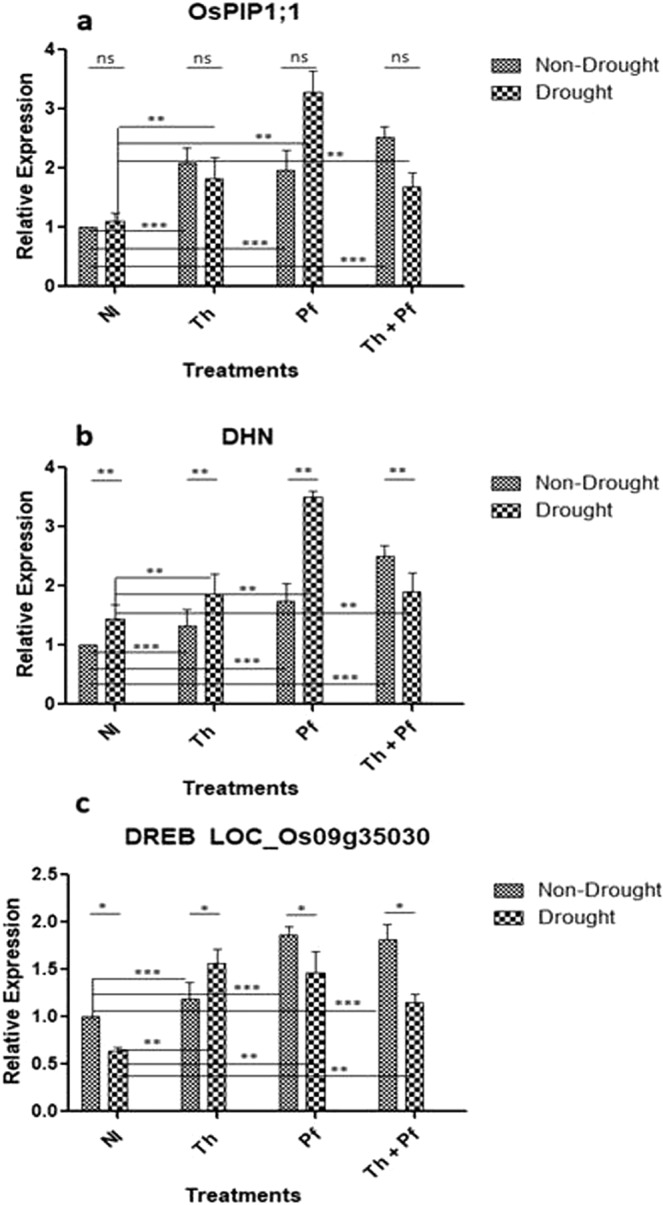
Effect of microbial inoculation and drought stress on the expression of *OsPIP1*(**a**), *DHN* (**b**) and *DREB* (**c**) genes related to less water permeability and dehydration tolerance in rice. Significance values were determined using two-way ANOVA. n = 3; Data are shown as mean ± SEM for each sample; ns is non-significant.

We analysed gene expression of *OsPIP1;1*, a prominent representative of rice plasma-membrane protein gene family that regulates aquaporin^96^. The impact of inoculation on the expression of *OsPIP1;1* was statistically significant [F(3,16) = 12.34, *p*0.0002] but that of drought was non-significant [F(1,16) = 0.1953, *p*0.6644] (Fig. 7a, Supplementary Table 6). The interaction effect on the expression of this gene was statistically significant [F(3,16) = 6.054, *p*0.0059]. Microbial inoculation therefore, up-regulated *OsPIP1;1* of the PIP gene family in both the cohorts of stressed and non-stressed plants. *OsPIP1;1* is an important gene, the protein product of which is related to less water permeability in the plant cells^97^. We showed that microbial inoculation in plants growing normally (non-stress) led to up-regulation of *OsPIP1;1* gene. Within the cohort of stressed plants, maximum upregulation was observed in plants inoculated with *Pf* alone (Fig. 7a). The results indicate positive role of microbial inoculation in the modulation of *OsPIP1* gene, which regulates aquaporin, the water channel protein that mediates stress tolerance in rice plants.

Dehydrins (DHNs) play key role in responding to adaptation against abiotic stresses^98^. Microbial inoculation up-regulated the expression of DHN gene in rice plants grown under stressed and non-stressed conditions both (Fig. 7b). In the cohort of stressed plants, inoculation of *Pf* showed maximum *DHN* expression. Results of the two-way ANOVA for DHN gene expression indicated significant effects of watering regime [F(1,16) = 9.408, *p*0.0074] and microbial inoculation [F(3,16) = 13, *p*0.0001]. Interaction also had significant impact on DHN gene expression [F(3,16) = 7.799, *p*0.002] (Fig. 7b, Supplementary Table 6). In the dehydrating plant cells due to prolonged drought, over-expression of dehydrin genes in the cytoplasm and vicinity of plasma membrane is an important phenomenon^28,99,100^. The up-regulation of the gene protects structural and functional enzymes, proteins and nucleic acids during oxidative damage^101,102^ and enhances efficiency of crop plants against abiotic stresses^103^. The microbial inoculation was shown to facilitate high expression of *DHN* gene in rice to offer protection to vegetative tissues against dehydration and desiccation under challenged osmotic stress.

Dehydration responsive element binding (DREB) transcription factors improve abiotic stress tolerance in plants through regulation of stress-inducible gene expression^98,100,104^. The impact of microbial inoculation in rice plants for the expression of *DREB* gene was significant [F(3,16) = 14.71, *p* < 0.0001]. The effects of drought [F(1,16) = 7.527, *p*0.0144) and interaction [F(3,16) = 5.383, *p*0.0094) was also significant (Fig. 7c, Supplementary Table 6). These observations, together with the enzyme activity provided evidences to confirm that microbial inoculation modulates expression of stress responsive genes linked with dehydration. This further makes a clearer understanding on the activation of strategic molecular mechanisms meant for avoidance or adaptation against stress damage in rice due to microbial inoculation.

### Inoculants improved expression of genes encoding dismutation of superoxide radicals

**Figure 8:**
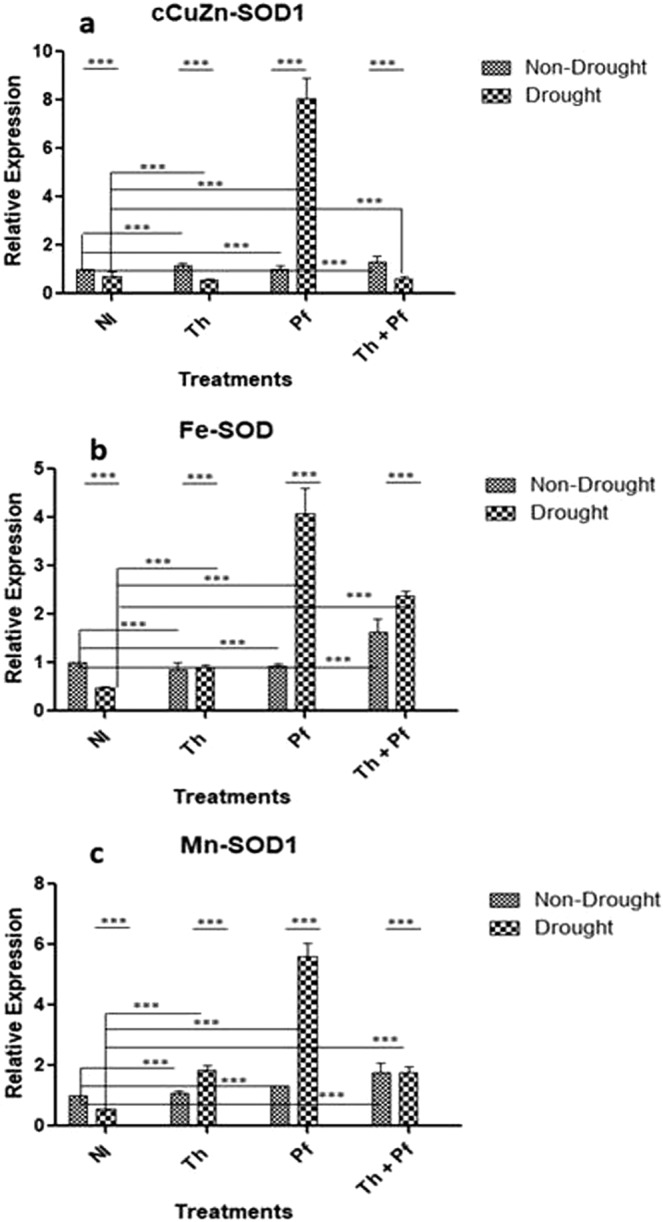
Effect of microbial inoculation on the expression of *CuZn-SOD* (**a**), *Fe-SOD* (**b**) and *Mn-SOD* (**c**) genes in the leaves of rice plants grown under drought and non-drought condition. Data are represented as mean ± SEM for each sample; Two-way ANOVA was performed to determine statistical significance; n = 3 for transcript analysis.

In plants, SOD constitutes the first line of defense against ROS-induced damage^82^. To gain insight into the expression of SOD gene group *CuZn-SOD* (localized to chloroplasts and cytosol), *Mn-SOD* (bound to mitochondria) and *Fe-SOD* (localized to chloroplast), their expression in rice grown under drought following microbial inoculation was assessed. On *CuZn-SOD*, the impact of all the three experimental factors, *viz*. drought [F(1,16) = 35.57, *p* < 0.0001], microbial inoculation [F(3,16) = 62.73, *p* < 0.0001) and interaction [F(3,16) = 67.17, *p* < 0.0001] was significant (Fig. 8a, Supplementary Table 7). On *Fe-SOD* gene also, the impact of microbial inoculation [F(1,16) = 32.01, *p* < 0.0001], watering regime [F(3,16) = 31.35, *p* < 0.0001] and interaction [F(3,16) = 28.02., *p* < 0.0001] was significant (Fig. 8b, Supplementary Table 7). Like *CuZn-* and *Fe-SOD*, the effects of drought [F(1,16) = 61.24], microbial inoculation [F(3,16) = 59.83] and interaction factor [F(3,16) = 52.86] on the expression of *Mn-SOD1* was also significant at *p* < 0.0001 (Fig. 8c). It was interesting that within the cohort of the three treatments of microbial inoculation in plants growing under stressed condition, inoculation of *Pf* bacteria showed high upregulation values for all the three genes (Fig. 8). Except for the DREB gene which showed maximum over expression in the cohort of non-stressed plants inoculated with *Pf* (Fig. 7c), inoculation of plants with the bacteria *Pseudomonas* alone showed consistently high expression values of *OsPIP1;1, DHN* and all the three isomorphs of SOD genes in the cohort of stressed plants (Figs. 7a,b and 8a–c). It was concluded that the over-expression of SOD gene isoforms leads to enhanced activity of SOD enzyme in rice plants grown under microbial inoculation and drought challenged condition. Presumably, the enhanced gene expression and subsequent enzyme activity level might have played an important role in reducing the deleterious impact of ROS in rice grown under stress.

### Microbial inoculation enhanced expression of genes encoding peroxidation of H_2_O_2_

**Figure 9:**
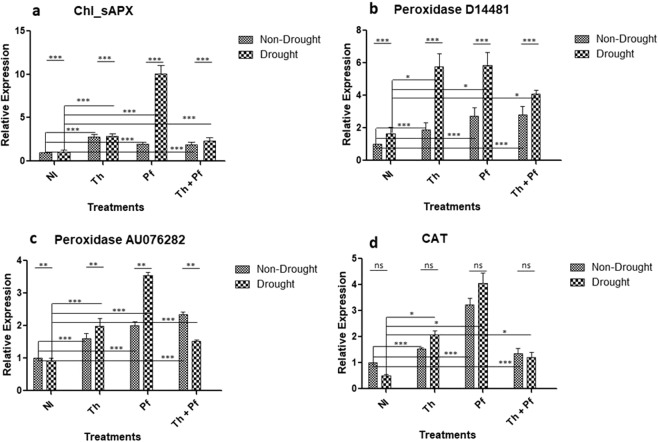
Impact of microbial inoculation on the expression of Chl_sAPX (**a**), peroxidase D14481 (**b**) peroxidase AU076282 (**c**) and Catalase (**d**) genes in rice plants grown under drought and non-drought conditions. Statistical significance was determined by two-way ANOVA; data are mean ± SEM; n = 3 for transcript analysis. ns is non-significant.

*APX* gene regulates ascorbate-glutathione (AsA-GSH) cycle that plays key role in the reduction of H_2_O_2_ to H_2_O^105,106^. Over-expression of APX gene in plants improves oxidative defense and offers tolerance to abiotic stress^105^. In the cohort of plants grown with stress and inoculated with *Th*, *Pf* and *Th* + *Pf*, single inoculation of *Pf* showed high overexpression of *APX* gene than *Th* or combined inoculation of *Th* + *Pf* (Fig. 9a). The impact of drought [F(1,16) = 46.30], microbial inoculation [F(3,16) = 45.21], interaction [F(3,16) = 38.55] was significant at *p* < 0.0001 (Fig. 9a, Supplementary Table 8). The bacterial inoculant *Pf* showed maximum over-expression of *APX* gene in plants under drought condition than *Th* or doubly inoculation of *Th* + *Pf*. Inoculating plants with microbial inoculants enhanced expression of the peroxidase (*PO*) genes (*PO* D14481 and *PO* AU076282) in rice. Within the cohort of stressed and non-stressed plants, inoculation resulted in enhanced over-expression than the control. Maximum over-expression was again recorded in plants grown under stressed conditions and inoculated with *Pf* (Fig. 9b,c). The impact of watering regime on the expression of the gene *PO* D14481 was significant [F(1,16) = 35.54, *p* < 0.0001] and similar was the effect of microbial inoculation [F(3,16) = 12.27, *p*0.0002]. The interaction impact on expression of this gene was also significant [F(3,16) = 4.150, *p*0.0236] (Fig. 9b; Supplementary Table 8). Likewise, the impact of drought [F(1,16) = 8.962, *p*0.0086], microbial inoculation [F(3,16) = 73.23, *p* < 0.0001] and interaction of drought and inoculation factor [F (3,16) = 32.28, *p* < 0.0001] on another peroxidase gene *PO* AU076282 was significant (Fig. 9c, Supplementary Table 8). The effect of the inoculation of bacterial inoculant *Pf* on the expression of *PO* AU076282 gene in plants grown under drought was maximum than *Th* or doubly inoculation of *Th* + *Pf*. Results indicated that microbial inoculation helped rice plants in over-expressing peroxidases and the inoculation of *Pseudomonas* was invariably instrumental in highest over-expression of these genes. Peroxidases are the key genes in regulating ROS scavenging and thus, their over-expression in rice can have protective role in plants exposed to drought.

Over-expression of *CAT* gene enhances oxidative defense response in plants^107^. Inoculation of rice grown under drought condition with *Pf* resulted in highest level of expression of *CAT* gene in the cohort of drought stressed and inoculated plants (Fig. 9d). The impact of drought on the gene expression was non-significant [F(1,16) = 1.898, *p*0.1873]. The effects of microbial inoculation [F(3,16) = 81.48, *p* < 0.0001] and interaction (drought *vs*. inoculation) [F(3,16) = 4.739, *p*0.0150] were significant (Fig. 9d; Supplementary Table 8). The results strongly suggested that microbial inoculation had a positive role in the over-expression of the genes linked with the peroxidation of H_2_O_2_ in the plants challenged with the drought. Invariably, the effect of inoculation of *Pseudomonas* substantially enhanced *APX*, *PO* and *CAT* gene expression in plants grown under stressed condition. These modulations in gene expression may support improved drought tolerance in rice plants.

## Conclusion

We have shown that although drought suppressed growth of rice plants, as is evident from reduced shoot and root weight, microbial inoculation managed to reduce the impact of drought. There have been multi-pronged mechanisms utilized and adopted by the plants to mitigate and/or minimize the impact of drought if the plants were inoculated with the microbial species. Generation of ROS is a common phenomenon in plant cells under stressed conditions. We reported hyperaccumulation of H_2_O_2_ in rice leaves and the resultant hypersensitive cell death responses thereafter. Induced accumulation of the *PAL* gene transcripts and resultant activation of PAL enzyme facilitated higher accumulation of the phenylpropanoids that have strong ROS scavenging activity and might have helped plants to overcome oxidative burden created due to drought stress.With the activation of the antioxidant enzymes SOD, PO, APX and CAT, rice plants were supposed to minimize tissue damaging impact of high H_2_O_2_ levels. Over expression of all the isoforms of SOD, Cu-Zn SOD, Mn-SOD and Fe-SOD genes suggested that microbial inoculation helped plants activate SOD activity as first line of defence at various levels of cellular compartments strongly to overcome ROS burden. Microbial inoculation in plants further improved the activity of the enzymes PO, APX, GPX and GR that have also contributed in reducing ROS burden in the plants following drought challenge. We also observed enhanced regulation of less-water permeability-linked gene, *OSPiP1* that regulates aquaporin, drought-adaptation gene *DHN* and dehydration related *DREB* gene. Presumably, up-regulation of genes encoding phenylpropanoids, dismutation of superoxide radicals and peroxidation of H_2_O_2_ in microbe inoculated and drought challenged condition strongly contributed towards stress mitigation. Enhanced enzymatic and non-enzymatic antioxidant activities were thought to be the repercussions of the enhanced gene expression levels in microbial inoculated plants and have also helped in minimizing antioxidative load to overcome the oxidative stress. We further conclude that the physiological, biochemical and molecular mechanisms contributing to drought mitigation in rice following microbial interaction are multi-faceted, multi-channeled and interlinked. Results have shown that microbial inoculants have succeeded in improving intrinsic physiological and molecular capabilities of the plants mostly by reducing the damaging impact of the ROS, which was managed at multiple layers. Therefore, microbial inoculation could find an essential place in raising crops under abiotic stress conditions.

## Supplementary information

Supplementary Table 1.
