# Supplementary material for: Microbial inoculation in rice regulates antioxidative reactions and defense related genes to mitigate drought stress
Source: Sci Rep. 2020 Mar 16;10:4818. doi: 10.1038/s41598-020-61140-w (PMC7076003; doi:10.1038/s41598-020-61140-w)
Supplement: Supplementary file 1 — Supplementary Table 1. [file 41598_2020_61140_MOESM1_ESM.docx]

**Supplementary Table 1**

**Title Page for Supplementary Information**

Microbial inoculation in rice regulates antioxidative reactions and defense related genes to mitigate drought stress

Dhananjaya P. Singh^1*^, Vivek Singh^1^, Vijai K. Gupta^3^, Renu Shukla^1^, Ratna Prabha^1^, Birinchi K. Sarma^2^, Jai Singh Patel^2^

^1^ICAR-National Bureau of Agriculturally Important Microorganisms, Kushmaur, Maunath Bhanjan 275101, India.

^2^Department of Mycology and Plant Pathology, Institute of Agricultural Sciences, Banaras Hindu University, Varanasi 21005, India; email: birinchi_ks@yahoo.com

^3^Department of Chemistry and Biotechnology, School of Science, Tallinn University of Technology, Akadeemia tee 15, 12618 Tallinn, Estonia, email: vijaifzd@gmail.com

*Corresponding author email: Dhananjaya.Singh@icar.gov.in; dpsfarm@rediffmail.com; Tel: +915472530125; +919415291703

Supplementary Table 1.

| Response | Source | *df* | MS | *F* | *p* |
| --- | --- | --- | --- | --- | --- |
| H_2_O_2_ Production | Drought | 1 | 44400 | 296.9 | **<0.0001** |
|  | Inoculation | 3 | 16770 | 112.1 | **<0.0001** |
|  | Drought*Inoculation | 3 | 1254 | 8.388 | **0.0002** |
|  | Error | 40 | 149.5 |  |  |

Supplementary Table 2.

| Response | Source | *df* | MS | *F* | *p* |
| --- | --- | --- | --- | --- | --- |
| Total polyphenol content | Drought | 1 | 3823000 | 549.2 | **<0.0001** |
|  | Inoculation | 3 | 985300 | 141.5 | **<0.0001** |
|  | Drought*Inoculation | 3 | 123700 | 17.77 | **<0.0001** |
|  | Error | 40 | 6962 |  |  |
| Phenylalanie ammonia-lyase activity | Drought | 1 | 97920 | 135.0 | **<0.0001** |
|  | Inoculation | 3 | 118200 | 163.0 | **<0.0001** |
|  | Drought*Inoculation | 3 | 12360 | 17.04 | **<0.0001** |
|  | Error | 40 | 725.5 |  |  |
| PAL gene | Drought | 1 | 15.83 | 102.5 | **<0.0001** |
|  | Inoculation | 3 | 6.498 | 42.08 | **<0.0001** |
|  | Drought*Inoculation | 3 | 1.820 | 11.79 | **0.0003** |
|  | Error | 16 | 0.1544 |  |  |

Supplementary Table 3.

| Response | Source | *df* | MS | *F* | *p* |
| --- | --- | --- | --- | --- | --- |
| Free radical scavenging activity (%) | Drought | 1 | 2650 | 151.7 | **<0.0001** |
|  | Inoculation | 3 | 521.4 | 29.85 | **<0.0001** |
|  | Drought*Inoculation | 3 | 90.03 | 5.154 | **0.0042** |
|  | Error | 40 | 17.47 |  |  |
| ABTS Activity  Inhibition (%) | Drought | 1 | 1469 | 96.15 | **<0.0001** |
|  | Inoculation | 3 | 180.2 | 11.80 | **<0.0001** |
|  | Drought*Inoculation | 3 | 13.23 | 0.8662 | 0.4666 |
|  | Error | 40 | 15.28 |  |  |
| Reducing power  (% increase) | Drought | 1 | 16330 | 639.8 | **<0.0001** |
|  | Inoculation | 3 | 1561 | 61.13 | **<0.0001** |
|  | Drought*Inoculation | 3 | 161.8 | 6.339 | **0.0013** |
|  | Error | 40 | 25.53 |  |  |
| Fe-ion Chelation (%) | Drought | 1 | 691.5 | 27.63 | **<0.0001** |
|  | Inoculation | 3 | 657.9 | 26.28 | **<0.0001** |
|  | Drought*Inoculation | 3 | 31.40 | 1.255 | 0.3029 |
|  | Error | 40 | 25.03 |  |  |

Supplementary Table 4.

| Response | Source | *df* | MS | *F* | *p* |
| --- | --- | --- | --- | --- | --- |
| Superoxide Dismutase activity | Drought | 1 | 86.97 | 52.30 | **< 0.0001** |
|  | Inoculation | 3 | 5.982 | 3.598 | **0.0216** |
|  | Drought*Inoculation | 3 | 0.09009 | 0.05417 | 0.9832 |
|  | Error | 40 | 1.663 |  |  |
| Glutathione reductase activity | Drought | 1 | 1.078 | 147.2 | **< 0.0001** |
|  | Inoculation | 3 | 0.3229 | 44.07 | **< 0.0001** |
|  | Drought*Inoculation | 3 | 0.09131 | 12.46 | **< 0.0001** |
|  | Error | 40 | 0.007326 |  |  |

Supplementary Table 5.

| Response | Source | *df* | MS | *F* | *p* |
| --- | --- | --- | --- | --- | --- |
| PO activity | Drought | 1 | 5.309 | 239.6 | **< 0.0001** |
|  | Inoculation | 3 | 0.2650 | 11.96 | **< 0.0001** |
|  | Drought*Inoculation | 3 | 0.01346 | 0.6073 | 0.6141 |
|  | Error | 40 | 0.02216 |  |  |
| Catalase activity | Drought | 1 | 11.05 | 379.9 | **<0.0001** |
|  | Inoculation | 3 | 0.8850 | 30.42 | **<0.0001** |
|  | Drought*Inoculation | 3 | 0.01825 | 0.6272 | 0.6017 |
|  | Error | 40 | 0.02909 |  |  |
| GPX | Drought | 1 | 1.302 | 423.7 | **<0.0001** |
|  | Inoculation | 3 | 0.07145 | 23.26 | **<0.0001** |
|  | Drought*Inoculation | 3 | 0.4732 | 15.40 | **<0.0001** |
|  | Error | 40 | 0.003072 |  |  |
| APX | Drought | 1 | 0.5471 | 59.45 | **<0.0001** |
|  | Inoculation | 3 | 7.150 | 776.9 | **<0.0001** |
|  | Drought*Inoculation | 3 | 0.3081 | 33.47 | **<0.0001** |
|  | Error | 40 | 0.009203 |  |  |

Supplementary Table 6.

| Response | Source | *df* | MS | *F* | *p* |
| --- | --- | --- | --- | --- | --- |
| OsPIP1;1 | Drought | 1 | 0.04085 | 0.1953 | 0.6644 |
|  | Inoculation | 3 | 2.580 | 12.34 | **0.0002** |
|  | Drought*Inoculation | 3 | 1.266 | 6.054 | **0.0059** |
|  | Error | 16 | 0.2091 |  |  |
| DHN | Drought | 1 | 1.710 | 9.408 | **0.0074** |
|  | Inoculation | 3 | 2.383 | 13.00 | **0.0001** |
|  | Drought*Inoculation | 3 | 1.417 | 7.799 | **0.0020** |
|  | Error | 16 | 0.1817 |  |  |
| DREB LOC_Os09g35030 | Drought | 1 | 0.4104 | 7.527 | **0.0144** |
|  | Inoculation | 3 | 0.8019 | 14.71 | **<0.0001** |
|  | Drought*Inoculation | 3 | 0.2935 | 5.383 | **0.0094** |
|  | Error | 16 | 0.05452 |  |  |

Supplementary Table 7.

| Response | Source | *df* | MS | *F* | *p* |
| --- | --- | --- | --- | --- | --- |
| cCuZn-SOD1 | Drought | 1 | 11.41 | 35.57 | **<0.0001** |
|  | Inoculation | 3 | 20.12 | 62.73 | **<0.0001** |
|  | Drought*Inoculation | 3 | 21.55 | 67.17 | **<0.0001** |
|  | Error | 16 | 0.3208 |  |  |
| Fe-SOD | Drought | 1 | 4.410 | 31.35 | **<0.0001** |
|  | Inoculation | 3 | 4.502 | 32.01 | **<0.0001** |
|  | Drought*Inoculation | 3 | 3.941 | 28.02 | **<0.0001** |
|  | Error | 16 | 0.1407 |  |  |
| Mn-SOD1 | Drought | 1 | 8.062 | 61.24 | **<0.0001** |
|  | Inoculation | 3 | 7.876 | 59.83 | **<0.0001** |
|  | Drought*Inoculation | 3 | 6.959 | 52.86 | **<0.0001** |
|  | Error | 16 | 0.1317 |  |  |

Supplementary Table 8.

| Response | Source | *df* | MS | *F* | *p* |
| --- | --- | --- | --- | --- | --- |
| Chl_sAPX | Drought | 1 | 28.17 | 46.30 | **<0.0001** |
|  | Inoculation | 3 | 27.50 | 45.21 | **<0.0001** |
|  | Drought*Inoculation | 3 | 23.45 | 38.55 | **<0.0001** |
|  | Error | 16 | 0.6084 |  |  |
| Peroxidase D14481 | Drought | 1 | 29.88 | 35.54 | **<0.0001** |
|  | Inoculation | 3 | 10.31 | 12.27 | **0.0002** |
|  | Drought*Inoculation | 3 | 3.490 | 4.150 | **0.0236** |
|  | Error | 16 | 0.8409 |  |  |
| Peroxidase AU076282 | Drought | 1 | 0.4073 | 8.962 | **0.0086** |
|  | Inoculation | 3 | 3.328 | 73.23 | **<0.0001** |
|  | Drought*Inoculation | 3 | 1.467 | 32.28 | **<0.0001** |
|  | Error | 16 | 0.04545 |  |  |
| CAT Gene | Drought | 1 | 0.2209 | 1.898 | 0.1873 |
|  | Inoculation | 3 | 9.486 | 81.48 | **<0.0001** |
|  | Drought*Inoculation | 3 | 0.5518 | 4.739 | **0.0150** |
|  | Error | 16 | 0.1164 |  |  |

*p* values in bold are significantly different
